# Plant origin and irrigation influence floral resource value and pollinator attraction to ornamental plants

**DOI:** 10.7717/peerj.20906

**Published:** 2026-03-12

**Authors:** Joanna J. Silva, Taehoon Kim, Kevin Begcy, Xavier Martini, Gary Knox, Rachel E. Mallinger

**Affiliations:** 1 Department Entomology and Nematology, University of Florida, Gainesville, Florida, United States; 2 Department Environmental Horticulture, University of Florida, Gainesville, FL, United States; 3 Department Entomology and Nematology, University of Florida, Quincy, Florida, United States; 4 Department Environmental Horticulture, University of Florida, Quincy, Florida, United States

**Keywords:** Native plants, Non-native plants, Irrigation, Floral resources, Pollinators

## Abstract

Irrigation and plant origin are key factors influencing plant growth, development, and reproductive strategies. These same factors may also affect the floral resource value of plants to pollinators, influencing the efficacy of pollinator plantings for conservation or enhancement. While these factors have been studied separately, few studies have examined how plant origin influences plant responses to irrigation for floral traits such as nectar and pollen. In this study, we evaluated 10 native and 10 non-native plants to Florida under two different irrigation treatments and measured floral density, floral display, floral resources (nectar and pollen), and pollinator visitation rates and diversity in an open field study. There was strong to very strong evidence that native plants produced higher floral densities and displays, along with greater amounts of pollen per plot, and attracted a higher number and diversity of pollinators as compared to non-native plants. Additionally, there was weak to moderate evidence that fully irrigated plants produced higher nectar volumes and pollen quantities per plot as well as pollen with higher protein content as compared to partially irrigated plants. However, there was no evidence that native and non-native plants responded differently to irrigation treatment. Additionally, individual pollinator guilds responded differently to origin and irrigation, highlighting the complexity of plant-pollinator interactions. We conclude that native plants consistently produce more flowers and attract a greater number and diversity of pollinators while irrigation affects the production and resource value of pollen and nectar. These findings emphasize the importance of considering both plant origin and irrigation on resource availability of flowers when selecting pollinator-friendly plants under varying climates and management regimes.

## Introduction

Global declines have been reported for numerous pollinator taxa (insects, birds, and bats), which in turn decrease the worldwide provisioning of pollination services (Goulson et al., 2015; IPBES, 2016). The decline in pollinators is attributed to multiple interacting drivers including pathogens, pesticides, urbanization, habitat fragmentation, and climate change (Steffan-Dewenter et al., 2002; Cane et al., 2006; Potts et al., 2010; Baldock et al., 2015; Goulson et al., 2015; Potts et al., 2016). Climate change impacts pollinators at different levels; for example, it can cause range shifts in plants and/or pollinators leading to spatial mismatches in plant-pollinator interactions (Memmott et al., 2007; Potts et al., 2010, 2016; Burkle, Marlin & Knight, 2013; Gérard et al., 2020), and it can lead to changes in pollinator physiology and phenology, further impacting their interactions with plants including potential morphological and phenological mismatches (Hegland et al., 2009; Kudo & Ida, 2013; Gérard et al., 2020). Additionally, climate change and its associated changes in temperature and precipitation can impact plant vegetative and floral traits, including the quality and quantity of floral resources for pollinators (Carminati & Javaux, 2020; Kuppler & Kotowska, 2021).

Changes in water availability, including fluctuations in precipitation and more frequent and severe droughts, directly affect plant performance and can disrupt plant-pollinator interactions by altering pollinator resources (Gallagher & Campbell, 2017). In periods of drought, plants sensitive to water limitation may alter their floral traits and rewards in different ways (Descamps, Quinet & Jacquemart, 2021). For example, water limitation can result in reductions in the total amount and quality of nectar and pollen and/or in alterations to floral signals (visual and olfactory cues) that may affect pollinator visitation rates and in turn, compromise plant reproductive success (Carroll, Pallardy & Galen, 2001; Martini & Stelinski, 2017; Phillips et al., 2018; Rering et al., 2020; Descamps et al., 2021; Kuppler & Kotowska, 2021; Kuppler et al., 2021; Jaworski et al., 2022). Specifically, water limitation can reduce floral abundance, flower size (Carroll, Pallardy & Galen, 2001; Phillips et al., 2018; Kuppler et al., 2021), nectar volume (Carroll, Pallardy & Galen, 2001; Descamps et al., 2018; Phillips et al., 2018; Rering et al., 2020; Jaworski et al., 2022), and also alter nectar sugar concentration (Descamps et al., 2018; Phillips et al., 2018) including reductions in the proportion of sucrose in nectar (Rering et al., 2020). Water limitation along with changes in temperature might also reduce pollen viability (Su et al., 2013; Descamps et al., 2018), pollen quantity (Waser & Price, 2016; Arathi & Smith, 2023; Quinanzoni, Marcolet & Michelot-Antalik, 2024), and pollen nutritional values (polypeptides, protein content or lipid concentration) (Russo et al., 2019; Descamps et al., 2021).

Floral rewards including nectar and pollen represent a significant source of nutrition for insect pollinators. While nectar provides the primary source of carbohydrates for a variety of insect pollinators, pollen is the principal source of proteins and lipids for pollinators and is also consumed by some other insect groups (*e.g*., butterflies and beetles) (Majewska et al., 2018; Vaudo, Dyer & Leonard, 2024). Variation in pollen availability and quality can thus have significant effects on pollinator fitness; for example, pollen protein content directly affects reproduction and larval development in bees and higher pollen lipid content can increase bee larval size and growth (Vaudo et al., 2016). The quantity and quality of both nectar and pollen rewards can also influence pollinator recruitment. Alterations in nectar volume have been shown to affect flower visitation by insect pollinators and birds (Benitez-Vieyra et al., 2014; Majewska et al., 2018; Mallinger & Prasifka, 2017) and alterations in pollen availability or nutritional quality can similarly impact recruitment by affecting the foraging efficiency and preferences of pollinators, as well as their reproductive success and offspring development (Russo et al., 2019).

Habitat loss due to urbanization, and intensive agricultural practices is an additional major driver of pollinator declines, as it can lead to a reduction in the abundance and diversity of floral resources for pollinators (Dicks et al., 2016). A potential remedy to habitat and resource loss is to enhance urban green spaces with flowering plants for pollinators, though the value of these green spaces for pollinators will depend on plant species selection (Munschek et al., 2023) and management such as mowing frequency. Researchers have found that bee abundance and/or richness in urban gardens with flowering plants was comparable to, or even higher than, other rural habitats including nature reserves, parks, or other greenspaces (Baldock et al., 2019; Theodorou et al., 2017). However, not all flowering plants within urban gardens have the same value for pollinators; of plants commercially labeled as pollinator-friendly, some show limited pollinator attraction (Garbuzov & Ratnieks, 2014; Salisbury et al., 2025). Additionally, while many plants are labeled and sold as pollinator-friendly outside of their native range (Nota et al., 2024), their value to native pollinators will likely vary across pollinator guilds (Seitz, vanEngelsdorp & Leonhardt, 2020).

Specialist pollinators, such as specialist bees, are particularly sensitive to plant origin and identity while generalist pollinators may be able to take advantage of a greater variety of plants. As such, studies have found an increase in generalist pollinators in highly altered and developed habitats while specialist pollinators tend to decrease within urban areas that contain a high proportion of non-native plants (Burkle, Marlin & Knight, 2013; Baldock et al., 2019; Baldock, 2020; Staab, Pereira-Peixoto & Klein, 2020). Additionally, while some studies suggest a positive relationship between the abundance of native plants and the diversity of pollinators in gardens (Rollings & Goulson, 2019; Anderson et al., 2022; Palmersheim et al., 2022), other studies have found the opposite, or present mixed results (Majewska et al., 2018; Staab, Pereira-Peixoto & Klein, 2020; Seitz, vanEngelsdorp & Leonhardt, 2020). Explanations for such variable results may include whether non-native plants are native to nearby areas or naturalized to the study region *vs* native to regions further away. Additionally, the discrepancies may be due to the large variation in resource quantity and quality across cultivated plants. For example, some cultivated plants are bred to be sterile, producing little or no pollen for pollinators (Fetridge, Ascher & Langellotto, 2008; Matteson, Ascher & Langellotto, 2008; Frankie et al., 2019; Wilson & Deng, 2023). Finally, whether or not non-native plants are attractive or rewarding to generalist pollinators may depend on if they are functionally similar to native plants within the area including in their flower morphology or reward composition (Hayes et al., 2025).

Understanding how inputs such as water, pesticides, or fertilizer influence the floral resources provided by ornamental plants remains an important research question for pollinator-friendly gardening. Because native plants are adapted to the environmental conditions of a given area, they have been shown to exhibit higher stress tolerance to the abiotic conditions of the natural environment such as drought (Frost et al., 2025) or dry soils conditions (Gibson et al., 2019) and may thus provide more resources for pollinators under reduced inputs. However, while non-native plants might not be adapted to local conditions (Johnson, Fetters & Ashman, 2017), previous research has shown that they can also be tolerant to variable inputs, including showing no response to different irrigation levels and thus high tolerance to variable soil moisture levels (Shober et al., 2010). Other studies show that many non-native plants become naturalized or even invasive in new ranges, adapting to the local environmental conditions including water availability (Memmott & Waser, 2002; Razanajatovo et al., 2015). However, most of these studies tend to focus on how inputs affect vegetative traits, or floral density, and there is little information how varying inputs including water affect nectar and pollen production of native *vs* non-native plants. Selecting species that are well matched to local environmental conditions, and can perform well under limited inputs, is key for sustainable pollinator gardening.

This study aimed to assess the effects of plant origin and water inputs, and their interaction, on floral rewards including pollen and nectar and subsequently on pollinator recruitment. The specific objectives were to assess the effects of plant origin (native *vs* non-native) and irrigation (full and partial) on: (1) flower density and display size, (2) nectar volume, pollen quantity, and pollen protein content, and (3) pollinator visitation rates and the diversity of pollinators attracted. Currently, many home and urban gardens incorporate a mix of native and non-native ornamental species, many of which are also highly cultivated (Seitz, vanEngelsdorp & Leonhardt, 2020; Zinnen & Matthews, 2022), which could affect both their resource value and their dependence on inputs for optimal performance. Additionally, in our study region in Florida, plants may experience water stress due to seasonal droughts, limited irrigation, and/or lack of adaptation to the climate (Boyer et al., 2014; Matrazzo & Bissett, 2020; Ruiz et al., 2020). While many studies have examined plant origin and irrigation separately, our study aimed to determine how plant origin influences responses to water inputs for a variety of floral and pollinator traits. We hypothesized that Florida native plants would outperform non-native plants under reduced irrigation by providing more floral resources and attracting more pollinators. Additionally, we hypothesized that native plants would recruit a higher abundance and diversity of many pollinators compared to non-native plants, but non-native plants may attract certain generalist pollinators.

## Methods

### Plant species

We selected twenty ornamental plant species, organized into ten native to Florida and ten non-native to Florida congeneric pairs, following the criteria established in Silva et al. (2024). These criteria included availability in nurseries, suitability for ornamental landscapes, attractiveness to pollinators, a broad range of flower colors and morphologies, and complementary blooming periods throughout the year (Florida-Friendly Landscaping™ Program, 2024). We also paired native and non-native plants at the genus level to ensure that differences in pollinator recruitment could be attributed to plant origin rather than significant differences in floral traits. Previous studies that do not control for trait similarity make it challenging to separate the effects of plant origin from floral trait variation on pollinator recruitment.

Briefly, the ten native species selected were: *Bidens alba* (L.) DC, *Conradina grandiflora* Small, *Coreopsis laevenworthii* L., *Gaillardia pulchella* Foug., *Hibiscus grandiflorus* Michaux, *Ilex glabra* L., *Monarda punctata* L., *Salvia azurea* Michx. ex Lam., *Scutellaria arenicola* L., and *Viburnum obovatum* Walter. The ten non-native counterparts were: *Bidens ferulifolia*, *Salvia officinalis*, *Coreopsis* $\times$ ‘Jethro Tull’, *Gaillardia* $\times$ *grandiflora*, *Hibiscus syriacus*, *Ilex cornuta* Lindl., *Monarda didyma*, *Salvia longispicata* $\times$ *S. farinacea*, *Scutellaria javanica* Jungh, and *Viburnum suspensum* Lindl. Detailed descriptions of categorization, plant habits (woody and herbaceous), flower color, and drought tolerance of the species are provided in Table S1. Full botanical descriptions were previously published in Silva et al. (2024).

### Experimental design and plant management

**Figure 1:**
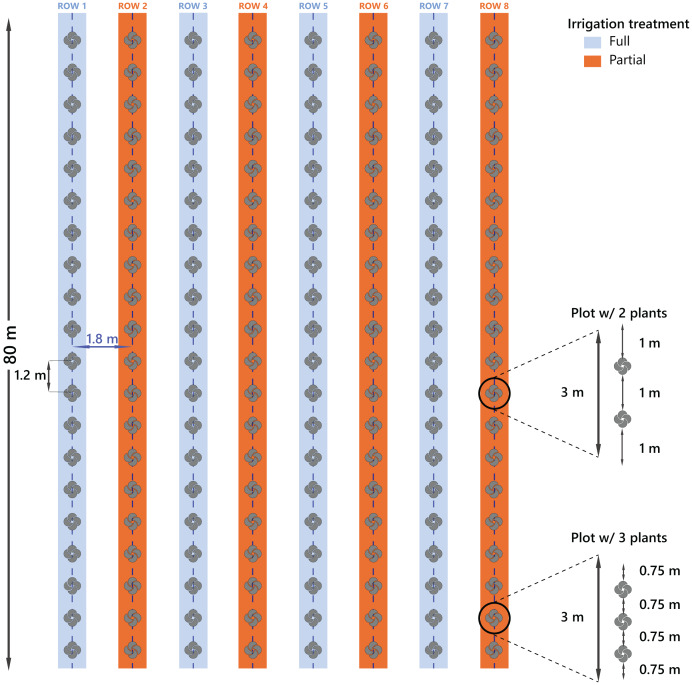
Experimental design showing eight beds (rows) per site, with four beds assigned to each irrigation treatment (Full or Partial). All twenty plant species were randomly distributed within each bed. Plots containing herbaceous species included three individuals per plot, whereas plots with woody species included two plants. The design was replicated at two sites: PSREU Citra, FL and NFREC, Quincy, FL.

The study was conducted from February 2021 to March 2023 at two UF/IFAS research centers in Florida. North Florida Research and Education Center (NFREC; Quincy, FL) and the Plant Science Research and Education Unit (PSREU; Citra, FL). Site-specific environmental data, including soil characteristics and climate conditions, are detailed in Silva et al. (2024). Plots in these two locations were prepared identically. At each site, we established eight slightly raised beds, which were prepared by disking and then covered with commercial-grade black landscape fabric following standardized protocols (Kalaman et al., 2022a; Silva et al., 2024; Wilson & Knox, 2006). The plots were planted with either two woody or three herbaceous individuals, spaced according to horticultural recommendations (Scheiber et al., 2007) (Fig. 1). Each bed contained 20 individual plots, with one plant species randomly assigned to each plot, which resulted in all 20 species (ten native and ten non-native) being represented within each bed.

Initially, all beds were drip irrigated daily for four weeks after planting to allow establishment as previously described (Silva et al., 2024). After this period, four of the eight beds at each site continued to receive full irrigation (2 h per day), while the remaining four beds were irrigated according to a volumetric soil moisture threshold of 10% using a SMRT-Y soil moisture sensor kit (Rainbird Inc., Tucson, AZ, USA). The sensors were installed at a 45^∘^ angle between two plots at the center of the beds, and they measured the soil moisture at the top 0.15–0.25 m of the bed following Zotarelli et al. (2011). Each bed was assigned to one of two irrigation treatments, resulting in four beds per irrigation treatment at each site (Fig. 1). The center of the beds where irrigation lines were installed were separated by 1.8 m to ensure independent water levels across beds (Fig. 1).

These two treatments represented full and partial irrigation, with full irrigation following typical landscape management in Florida and partial irrigation designed to provide supplemental water in periods of significant water limitation without compromising plant survival. The 10% threshold was chosen to be below manufacturer guidelines (recommended threshold 12%) but still allowing for plant survival, and based on previous studies in Florida in which the reduced irrigation level was set at 10–12% (Zotarelli et al., 2011). While Florida generally receives high precipitation annually, there are seasonal periods of significant water limitation, which combined with high solar irradiance and poor soil water holding capacity, result in water-use efficiency being a top priority for the landscaping and garden industries (Ferrarezi, Nogueira & Zepeda, 2020). Plant survival across years was high, although a few individuals per species were replaced at the start of the second year to maintain consistent plot density (see Silva et al., 2024).

### Floral resources

**Floral density** To evaluate flower density per plot across treatments, each plot was visited monthly over a 2-year period (March 2021–March 2023) and at both sites to count flowers. For species in the Asteraceae family (*Bidens*, *Coreopsis*, and *Gaillardia*), each capitulum (flower head) was counted as a single floral unit (Kalaman et al., 2022b; Silva et al., 2024). For species in the Lamiaceae family (*Conradina, Monarda, Salvia*, and *Scutellaria*), with more than thousands of individual flowers per plot arranged in inflorescences, we counted the number of inflorescences per plant and multiplied this by the average number of individual flowers per inflorescence counted across five inflorescences to obtain the estimated flower number per plant. For woody plants (*Hibiscus*, *Ilex*, *Viburnum*) we counted the total number of flowers per plant. To get flower density per plot, we multiplied the estimated number of flowers per plant by the number of plants per plot (2 or 3).

**Floral display** To evaluate the floral display area in each plot, we randomly selected 10 floral units per plot and measured the area of the individual floral units. For capitulate inflorescences, the floral unit was defined as the inflorescence (flower head) while for all other species, the unit was defined as a single flower. For radiate floral units (all families except Lamiaceae), the area of the floral unit was measured as the area of a circle $A = \pi {r^{2}}$. For the bilabiate floral units (Lamiaceae), we measured and multiplied the length of the lower lip and the length of the upper tube of the corolla (Benitez-Vieyra et al., 2014). We measured flower size within each plot 2–4 times per year during the species bloom period and over the 2 years of sampling period. Then, to determine the total floral display per plot per sampling day, we took the mean area per floral unit per plot per year (averaged across all individual measured floral units within the plot over the bloom period) and multiplied it by the number of flowers counted in each plot on each sampling day (Rowe et al., 2018, 2020, 2021).

**Nectar quantity** To evaluate nectar quantity, we measured nectar per flower in each plot twice during its bloom period in both years at the Northcentral FL site. On the day before sampling, 5-10 flowers per plot were randomly selected and bagged to exclude visitors for 24 h (Corbet, 2003; Hicks et al., 2016). The following day, we removed the bags and quantified the nectar volume for each of ten flowers per plot using microcapillary tubes (Dafni, 1992; Corbet, 2003; Hicks et al., 2016; Mallinger & Prasifka, 2017). We calculated the nectar volume per flower from our measurements as follows:

(1) $${{\rm {Length\;of\;the\;nectar\;column\;(mm)} } \over {\rm {Length\;of\;the\;pipette\;(mm)} \times {\mathrm{Calibrated\;volume\;of\;the\;pipette}} }}.$$

We additionally calculated the amount of nectar per plot per sampling date by multiplying the average nectar volume per flower (averaged across the flowers and time points sampled per plot per year) by the floral density per plot on each individual sampling date. For plots in North FL, nectar availability at the plot level per sampling day was estimated by multiplying floral density per plot on each sampling day with the average nectar volume per flower per plant species and irrigation treatment from Northcentral FL. This approach assumes that nectar volume per flower is a more stable attribute of a plant species as compared to flower abundance, which is highly influenced by site characteristics, and that relative differences in nectar volume per flower among different plant species are relatively consistent across sites even if overall nectar production is affected by site characteristics. This approach has been adopted by numerous past studies due to limitations in measuring nectar across all landscapes or study sites (Baude et al., 2016; Tew et al., 2021; Langlois et al., 2025).

**Pollen quantity** To assess pollen quantity, we collected five pre-dehiscent flowers in each plot at the Northcentral FL site during peak bloom and placed them in 50 ml centrifuge tubes with water in the laboratory next to natural light and allowed them to dehisce. We then randomly selected three of these five flowers for pollen quantification. For each selected flower, after dehiscence, we slightly crushed anthers to release all pollen grains into 1.5 mL microcentrifuge tubes with 1 mL of 70% ethanol and stored them at 4 ^∘^C for posterior analysis. The three samples were then vortexed for 15 s. From each sample, four standardized volume aliquots of 20 ${\rm\mu}$L were separately pipetted onto a Daigger Scientific® hemocytometer (Buffalo Grove, IL) and covered with a glass cover slide (Begcy et al., 2018; Kalaman et al., 2022b). Each 20 ${\rm\mu}$L aliquot was dispensed into two chambers of the hemocytometer, each holding 10 ${\rm\mu}$L. To count the pollen grains in each chamber, we used a hemocytometer with nine large square grids. The number of pollen grains was counted in the four corner quadrants and the central medium square of the square grid focusing the microscope at the 10 $\times$ objective. This process was repeated for the four aliquots per sample, vortexing the sample between aliquots to resuspend the pollen. To calculate the number of pollen grains per plot per sampling date, we took the average number of grains per flower per plot and then multiplied it by the floral density per plot per sampling date. Pollen grains per plot per sampling date at the North FL site was again determined by multiplying flower density per plot per sampling date with averaged pollen grains per flower per plant species and irrigation treatment measured in Northcentral FL following the same approach as for nectar described above and used in other studies (Wright et al., 2024). From the 20 species evaluated, we excluded pollenless flowers like *I. glabra* and its congener *I. cornuta* as the cultivars planted for those two species had only female flowers.

**Protein amount in pollen** To assess the total protein amount in pollen, we collected floral tissue samples from up to five pre-dehiscent flowers per plot during peak bloom; in Asteraceae species we collected five individual pre-dehiscent florets from five composite heads. All flowers were immediately placed in 1.5 mL Fisherbrand™ (Waltham, MA) premium microcentrifuge tubes with 1,000 ${\rm\mu}$L of 70% ethanol and stored in a −80 ^∘^C refrigerator until further analyses to prevent germination or pollen tube growth. The harvested samples were ground into a fine powder using liquid nitrogen. To prepare crude extracts for protein quantification, we utilized a urea buffer composed of the following components: 8 M Urea, 10 mM Tris-HCl (pH 6.8), 10% (v/v) glycerol, 1% (w/v) SDS, 5 mM DTT, and 1% (v/v) protease inhibitor cocktail designed for plant cell and tissue extracts (Sigma-Aldrich, St. Louis, MO, USA). The crude extracts were subjected to centrifugation at 12,000 g for 15 min at 4 °C, and the resulting supernatants were collected for further analysis. For the quantification of total protein content, we performed a Bradford assay using the Bio-rad Protein Assay Kit II (Bio-rad, Hercules, CA, USA). The absorbance of the samples was measured at 595 nm using an Epoch microplate spectrophotometer (BioTek, Winooski, VT, USA) to determine the total protein concentration. To calculate the total protein content, we normalized the total protein concentration by the weight of the tissue samples as previously described (Kalaman et al., 2022b).

### Pollinator recruitment

**Overall visitation rate** We assessed pollinator recruitment on the same day as we performed floral abundance surveys in each plot, including once per month for 2 years at both sites. On each sampling date, we conducted scanning counts in each plot twice per day during peak diurnal pollinator activity between 9:00 and 15:00 EST (Garbuzov & Ratnieks, 2014). Scanning counts consisted of an observer walking each of the eight beds per site and spending 1 min per plot scanning all flowers for visitors. During scanning counts, we counted every visit to a flower and recorded the visitor according to guild including honey bees (*Apis mellifera* L.), bumble bees (*Bombus* spp.), carpenter bees (*Xylocopa* spp, and *Ceratina* spp.), other bees, butterflies and moths (Lepidoptera), flies (Diptera), wasps, and other insects (including pollen beetles).

**Richness and diversity** To assess taxonomic diversity of insects visiting the plots, we collected specimens using individual vials after scanning counts. Collectors spent 5 min per plot for each plot on each observation day and collecting all insect visitors during that period. Collections were done separately from scanning counts to not disturb visitors when assessing visitation frequency. All samplings took place when climatic conditions were suitable with sunny to moderately overcast weather following Baldock et al. (2015). Then, all the specimens were labeled, pinned and identified to the lowest taxonomic level following taxonomic keys (Gibbs, 2011; Goulet & Huber, 1993; Kimsey, 2009; Triplehorn, Johnson & Borror, 2005) and the reference collections of the Mallinger Lab and the Florida State Collection of Arthropods (FSCA). All specimens are kept in the Mallinger Lab at the University of Florida.

### Statistical analysis

To assess how plant origin and irrigation interact to affect response variables, all below models had fixed effects of plant origin, irrigation, and their interaction. A summary of models including distributions, fixed, and random effects can be found in Table S2. For flower density per plot per sampling day, we used a generalized linear mixed model (GLMM) with a negative binomial distribution to account for overdispersion of count-based data. Plot nested within site, and year, were included as random effects to account for the repeated visits to a plot over time and variation across sites and years. For floral display per plot per sampling day, we accounted for skewed non-count data using a GLMM with a Tweedie distribution and with fixed and random effects as above.

For nectar volume per flower, we accounted for skewed non-count data using a GLMM with a Gamma distribution and included a random effect of plot to account for repeated sampling within a plot (across individual flowers). We also included person as a random effect to capture the variation between people when sampling nectar, which is sensitive to technique, and alterations can occur due to handling, contamination, or method (Corbet, 2003; Morrant, Schumann & Petit, 2009; Parachnowitsch, Manson & Sletvold, 2019), though we also ensured that sampler was not confounded with plant species or treatment, in this way we minimized the environmental variation that could cause measurement errors. For pollen quantity per flower, we accounted for overdispersion by using a GLMM with a negative binomial distribution and including a random effect of plot to account for repeated sampling within a plot across individual flowers. For standardized pollen protein content per flower ( ${\rm\mu}$L protein/mg flower tissue), we conducted a linear model with a normal distribution and with no random effects given that pollen was only collected at one site and one time point, and with flower tissues per plot lumped together for processing.

For total nectar volume per plot per sampling day (average nectar volume per flower* flower density per plot per day) we accounted for skewed non-count data using a GLMM with a Tweedie distribution, and including plot nested within site, and year, as random effects. For total pollen quantity per plot per sampling day (average pollen quantity per flower* flower density per plot per day) we accounted for skewed count data using a GLMM with a negative binomial distribution, and with plot nested within site as random effects.

To assess the visitation rate of the overall pollinator community (total pollinator visits per plot per sampling period), we used a GLMM with a negative binomial distribution and included plot nested within site, and year, as random effects. For each pollinator group separately (honey bees, bumble bees, carpenter bees, other wild bees, wasps, butterflies/moths, flies, and other insects) we used similar models with a negative binomial distribution for bumble bees, carpenter bees, other wild bees, wasps, butterflies/moths, and other insects, and zero-inflated negative binomial (ZINB) models for honey bees and flies. Plant species patterns for each pollinator guild are shown in Fig. S1.

For the diversity (Shannon Diversity Index) and species richness of flower-visiting insects per plot per sampling day, we generated GLMMs with a normal distribution for log-transformed Shannon Diversity Index and a negative binomial distribution for species richness to account for overdispersion. Plot nested within site, and year, were included as random effects as described above.

To determine the significance of fixed effects, all above-described models were subjected to an analysis of variance (ANOVA). We report the exact *p*-values and use the language of evidence to describe effects following Muff et al. (2022), where the evidence for an effect is described as very strong ( $p < 0.001$), strong ( $0.001 \le p < 0.01$), moderate ( $0.01 \le p\lt0.05$), weak ( $0.05 \le p\lt0.1$), and little or no evidence ( $P \ge 0.1$). This approach follows recent studies that have adopted the same framework (Bain et al., 2022; Kraus et al., 2023; Bernauer et al., 2024; Potts et al., 2024; Bhanda et al., 2025; Fiordaliso et al., 2025; Jain, Tang & Crall, 2025; Zanini et al., 2026). All analyses used the statistical software RStudio (Version 2023.06.2+561, ‘Mountain Hydrangea’, Boston, MA) including the packages glmmTMB for model building, car for ANOVA (Fox & Weisberg, 2019), emmeans for estimated means and standard errors (Lenth & Piaskowski, 2025), MASS (Venables & Ripley, 2002) and ggplot2 for visualization (Wickham, 2016). We also checked the model fit (residuals) for all models using the DHARMa (Hartig, 2024) package.

## Results

### Effects of origin and irrigation on floral density and display

**Figure 2:**
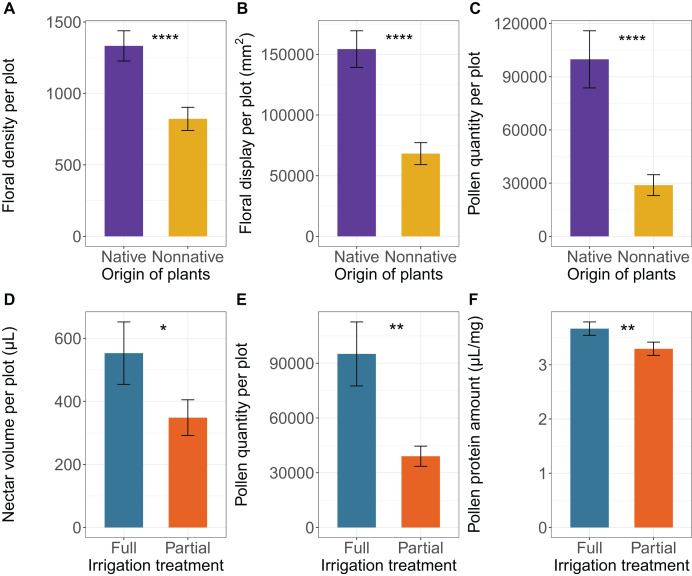
Effect of plant origin on (A) floral density per plot (number of individual floral units per plot), (B) floral display per plot (average size of floral unit * number of individual floral units per plot, mm^2^), and (C) pollen quantity per plot (average number of pollen grains per flower * flower density per plot), and effect of irrigation treatment on (C) nectar volume per plot (average nectar volume per flower * flower density per plot), (D) pollen quantity per plot, and (E) standardized protein amount in pollen. Means $\pm$ SE are shown for each variable, with **** indicating very strong evidence of an effect ( $p < 0.001$); *** strong evidence ( $0.001 < p < 0.01$) ** moderate ( $0.01 \le p < 0.05$), and * weak evidence ( $0.05 \le p < 0.1$).

There was very strong evidence that the origin of plants affected flower density (Table 1). Native plants from Florida produced 1.6 times more flowers than non-native plants (Fig. 2A). There was no evidence of irrigation treatment nor an interaction between origin and irrigation, indicating that native and non-native plants responded similarly to irrigation treatments (Table 1). There was also very strong evidence that the origin of plants affected floral display (Table 1). Native plants exhibited floral display 2.7 times larger than non-native plants (Fig. 2B). There was also no effect of irrigation or an interaction between origin and irrigation on floral display (Table 1).

**Table 1:** Effects of origin, irrigation and their interactions on floral density, display, and floral resources. We present the results of generalized linear models and estimated means along with standard errors (SE) for each evaluated variable, with **** indicating very strong evidence of an effect ( $p < 0.001$); *** strong evidence ( $0.001 < p < 0.01$) ** moderate ( $0.01 \le p < 0.05$), and * weak evidence ( $0.05 \le p < 0.1$).

Response and explanatory variable	Estimated means $\pm$ SE	$\chi$ ^2^	*p*-value
**Floral density**			
*Origin*		19.88	<0.001****
Native	1,117.30 $\pm$ 218.02****		
Non-native	525.81 $\pm$ 113.97****		
*Irrigation*		0.80	0.37
Full	823.88 $\pm$ 168.40		
Partial	713.07 $\pm$ 146.13		
*Origin $\times$ Irrigation*		0.02	0.89
**Floral display**			
*Origin*		35.36	<0.001****
Native	53,963.33 $\pm$ 21,585.91****		
Non-native	13,019.76 $\pm$ 5,355.70****		
*Irrigation*		1.00	0.32
Full	29,819.71 $\pm$ 12,078.09		
Partial	23,561.25 $\pm$ 9,550.48		
*Origin $\times$ Irrigation*		0.00	0.97
**Nectar volume per flower**			
*Origin*		0.33	0.57
Native	0.85 $\pm$ 0.33		
Non-native	0.71 $\pm$ 0.30		
*Irrigation*		2.21	0.14
Full	0.97 $\pm$ 0.37		
Partial	0.62 $\pm$ 0.25		
*Origin $\times$ Irrigation*		0.00	0.96
**Nectar volume per plot**			
*Origin*		0.58	0.45
Native	94.08 $\pm$ 61.42		
Non-native	77.52 $\pm$ 51.17		
*Irrigation*		3.64	0.05*
Full	108.65 $\pm$ 71.21*		
Partial	67.11 $\pm$ 44.14*		
*Origin $\times$ Irrigation*		0.01	0.94
**Pollen grains per flower**			
*Origin*		1.87	0.17
Native	240.05 $\pm$ 24.81		
Non-native	189.17 $\pm$ 27.69		
*Irrigation*		0.63	0.43
Full	228.16 $\pm$ 26.37		
Partial	201.07 $\pm$ 26.25		
*Origin $\times$ Irrigation*		0.27	0.60
**Pollen grains per plot**			
*Origin*		23.84	<0.001****
Native	84,021.36 $\pm$ 25,357.55****		
Non-native	27,298.76 $\pm$ 8,432.89****		
*Irrigation*		10.32	0.01**
Full	68,865.93 $\pm$ 21,026.96**		
Partial	33,306.44 $\pm$ 10,177.63**		
*Origin $\times$ Irrigation*		0.47	0.49
**Pollen protein**		***F-value***	
*Origin*		0.74	0.39
Native	3.41 $\pm$ 0.12		
Non-native	3.55 $\pm$ 0.13		
*Irrigation*		4.42	0.04**
Full	3.67 $\pm$ 0.12**		
Partial	3.29 $\pm$ 0.13**		
*Origin $\times$ Irrigation*		1.58	0.21

### Effects of origin and irrigation on floral resources

There was weak evidence that irrigation had an effect on nectar volume per plot (Table 1). Under full irrigation, plots produced 1.6 times more nectar than plots under partial irrigation (Fig. 2D). However, there was no evidence that nectar volume per flower was affected by irrigation (Table 1). There was also no evidence that the origin of plants had an effect on nectar volume per flower or per plot (Table 1), and no evidence of an interaction between irrigation and origin for nectar volume per flower or per plot (Table 1). There was also very strong evidence that pollen quantity per plot was affected by origin and moderate evidence that it was affected by irrigation, and with no evidence of an interaction between origin and irrigation (Table 1). Native plants produced 3.1 times more pollen per plot than non-native plants, and plots under full irrigation treatment had 2.1 times more pollen grains than partially irrigated plots (Figs. 2C, 2E). There was no evidence for effects of origin, irrigation, or their interaction, on pollen quantity at the per-flower level (Table 1). Finally, we found moderate evidence that irrigation affected pollen protein content per flower (Table 1): fully irrigated plants contained 1.1 times higher concentrations of protein per milligram of pollen than plants under partial irrigation treatment (Fig. 2F), and with no evidence of an effect of origin or an interaction between origin and irrigation on protein content per flower (Table 1).

### Influence of plant origin and irrigation on pollinator visitation rates

**Figure 3:**
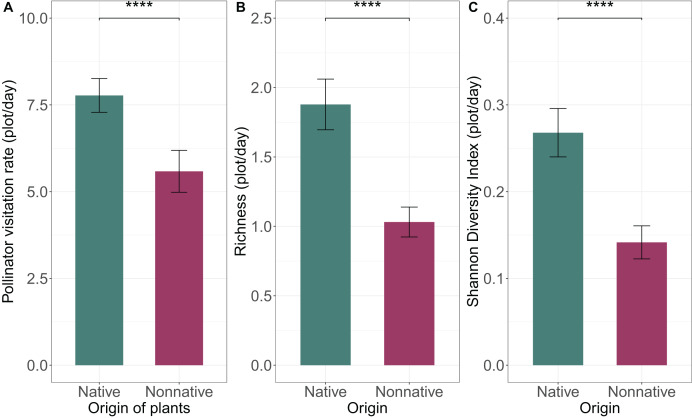
Effects of plant origin on (A) number of pollinator visits per plot observed per sampling day, (B) richness of pollinators per plot per sampling day, and (B) diversity (Shannon diversity index) of pollinators per plot per sampling day. Means $\pm$ SE are shown for each variable, with **** indicating very strong evidence of an effect ( $p < 0.001$); *** strong evidence ( $0.001 < p < 0.01$) ** moderate ( $0.01 \le p < 0.05$), and * weak evidence ( $0.05 \le p < 0.1$).

**Figure 4:**
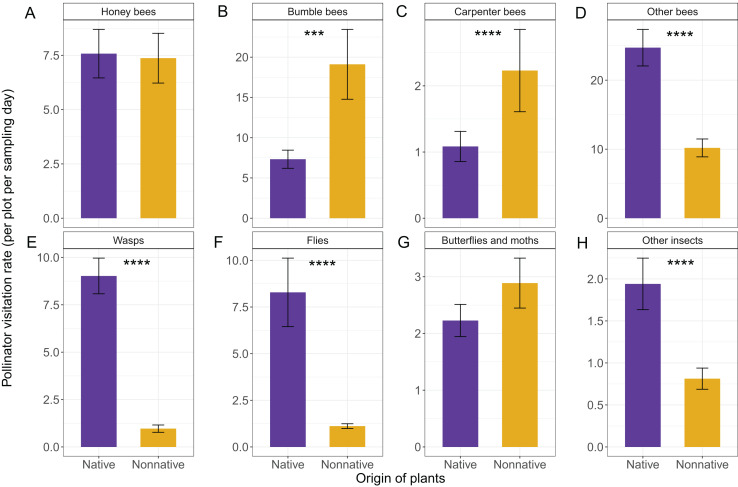
Effects of plant origin on the total number of visits of different pollinator groups per plot per sampling date including (A) Honey bees, (B) Bumble bees, (C) Carpenter bees, (D) Other bees, (E) Wasps, (F) Flies, (G) Butterflies and moths, and (H) Other insects. Means $\pm$ SE are shown for each variable, with **** indicating very strong evidence of an effect ( $p < 0.001$); *** strong evidence ( $0.001 < p < 0.01$) ** moderate ( $0.01 \le p < 0.05$), and * weak evidence ( $0.05 \le p < 0.1$).

**Figure 5:**
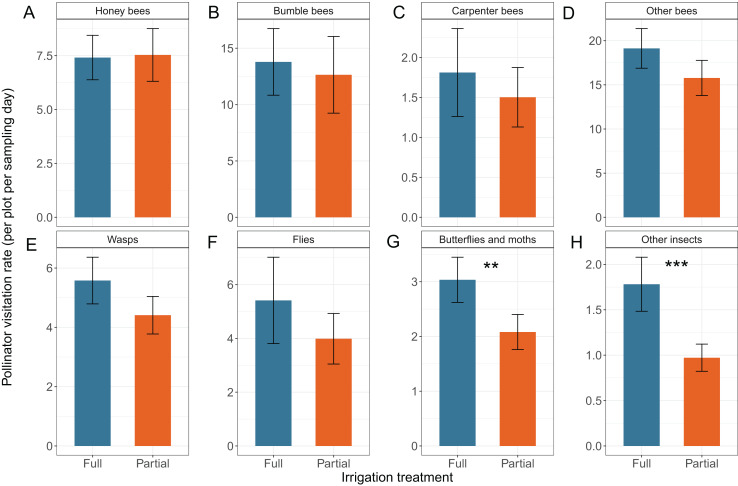
Effects of irrigation treatment on the total number of visits of different pollinator groups per plot per sampling date including (A) Honey bees, (B) Bumble bees, (C) Carpenter bees, (D) Other bees, (E) Wasps, (F) Flies, (G) Butterflies and moths, and (H) Other insects. Means $\pm$ SE are shown for each variable, with **** indicating very strong evidence of an effect ( $p < 0.001$); *** strong evidence ( $0.001 < p < 0.01$) ** moderate ( $0.01 \le p < 0.05$), and * weak evidence ( $0.05 \le p < 0.1$).

There was very strong evidence that total pollinator visits were influenced by plant origin ( ${X^{2}} = 22.55$, $p\lt0.001$), with native plants receiving more visits than non-native plants ( $7.77$ $\pm$ $0.49$, $5.58$ $\pm$ $0.60$ respectively) (Fig. 3A). However, there was no evidence that irrigation affected total pollinator visits ( ${X^{2}} = 1.55$, $p = 0.21$), and no evidence of an interaction between plant origin and irrigation ( ${X^{2}} = 0.15$, $p = 0.70$). Different pollinator groups responded differently to origin (Fig. 4) and irrigation (Fig. 5). Specifically, for honey bees, there was no evidence of an effect of plant origin ( ${X^{2}} = 0.06$, $p = 0.81$)(Fig. 4A), irrigation ( ${X^{2}} = 1.71$, $p = 0.19$) (Fig. 5A), or their interaction ( ${X^{2}} = 0.01$, $p = 0.90$). In contrast, there was strong or very strong evidence that bumble bees and carpenter bees preferred non-native plants over native ones ( ${X^{2}} = 9.76$, $p = 0.002$; ${X^{2}} = 11.75$, $p\lt0.001$, respectively) (Figs. 4B, 4C), but neither bumble bees nor carpenter bees showed evidence of a response to irrigation ( ${X^{2}} = 0.33$, $p = 0.56$; ${X^{2}} = 0.22$, $p = 0.64$, respectively) (Figs. 5B, 5C) or the interaction between origin and irrigation ( ${X^{2}} = 0.19$, $p = 0.66$; ${X^{2}} = 0.15$, $p = 0.70$, respectively). Other bees (primarily solitary bees) along with flies and wasps also showed very strong evidence of a preference for native plants ( ${X^{2}} = 11.70$, $p\lt0.001$; ${X^{2}} = 33.30$, $p\lt0.001$; ${X^{2}} = 19.57$, $p\lt0.001$, respectively) (Figs. 4D–4F). However, there was no evidence for an effect of irrigation ( ${X^{2}} = 0.91$, $p = 0.34$; ${X^{2}} = 0.35$, $p = 0.55$; ${X^{2}} = 0.15$, $p = 0.69$, respectively) (Figs. 5D–5F) or an interaction between origin and irrigation for any of these groups ( ${X^{2}} = 0.48$, $p = 0.49$; ${X^{2}} = 0.48$, $p = 0.49$; ${X^{2}} = 1.05$, $p = 0.31$, respectively). Alternatively, butterflies and moths showed moderate evidence of a preference for plants under full irrigation ( ${X^{2}} = 5.46$, $p = 0.02$) (Fig. 5G) but no evidence of a preference for plant origin ( ${X^{2}} = 0.07$, $p = 0.80$) (Fig. 4G) or an interaction effect ( ${X^{2}} = 0.08$, $p = 0.78$). Finally, there was very strong or strong evidence for effects of both plant origin and irrigation on visits by the group “other insects” (including pollen beetles) ( ${X^{2}} = 15.06$, $p\lt0.001$; ${X^{2}} = 6.73$, $p\lt0.01$, respectively), but no evidence of an interaction ( ${X^{2}} = 1.21$, $p = 0.27$). These insects preferred native and fully irrigated plants (Figs. 4, and 5H). Pollinator visitation rates by guild to individual plant species are additionally shown in Fig. S1.

### Pollinator diversity

We present a complete list of insects collected during the study and comprising each of the pollinator guilds (Table S3). We found very strong evidence that diversity and richness of insects collected in the plots were affected by the origin of plants ( ${X^{2}} = 12.00$, df = 1; $p\lt0.001$; ${X^{2}} = 11.33$, df = 1; $p\lt0.001$, respectively) but not irrigation ( ${X^{2}} = 1.21$, df = 1; $p = 0.27$; ${X^{2}} = 1.51$, df = 1; $p = 0.22$) or an interaction between origin and irrigation ( ${X^{2}} = 1.37$, df = 1; $p = 0.24$; ${X^{2}} = 1.38$, df = 1; $p = 0.24$). Native plants had higher pollinator richness and diversity than non-native plants (Figs. 3B, 3C).

## Discussion

Our study shows that plant origin and irrigation are factors that affect floral traits and resources including flower density and pollen and nectar production, which in turn affect pollinator recruitment. However, the direction and magnitude of effects differ across plant responses and pollinator guilds. For example, native plants consistently outperformed non-native plants in terms of floral density, floral display, and pollen quantity, and with strong to very strong evidence for such effects. In contrast, reduced irrigation reduced nectar and pollen quantity at the plot level and total protein content in pollen, albeit evidence for such effects was weak-moderate. However, contrary to our predictions, native plants did not perform better under reduced irrigation; instead, irrigation affected nectar and pollen quantity and quality similarly across both native and non-native plants. Pollinators responded differently to plant origin and irrigation, with many groups showing preferences for native plants with the exceptions of non-native bees (honey bees), generalist bees (carpenter and bumble bees) and butterflies/moths, the latter instead showing a preference for fully irrigated plants. These results highlight the importance of considering plant origin and irrigation as complementary factors in pollinator-friendly landscape management.

Plant origin had a strong influence on flower density and floral display, with native plants producing more flowers and larger displays than non-natives, aligning with previous findings reported by Kalaman et al. (2022a) and in line with our hypotheses. Pollinator visitation rates were also influenced by plant origin, with native plants attracting more total visits and a greater diversity of pollinators than non-native plants, results consistent with previous research findings (Salisbury et al., 2015; Fukase & Simmons, 2016; Palmersheim et al., 2022). The larger floral displays and higher flower density produced by native plants likely increased their visibility to pollinators, leading to higher pollinator visits (Seitz, vanEngelsdorp & Leonhardt, 2020).

Additionally, as we hypothesized, native plants attracted a higher diversity of pollinators including those in the group “other bees”, which are primarily solitary, wasps, flies, and other insects, the vast majority of which are native to our region and may be more specialized in their foraging. Although Florida host a few introduced solitary bees (*e.g*., *Megachile lanata, Centris nidita* and *Euglossa dilemma*), none of these species were detected in our sampling (see Table S3). Conversely, non-native honey bees showed no preference for plant origin (Hendriksma, Toth & Shafir, 2019). Additionally, despite being native to the area, bumble bees and carpenter bees preferred non-native plants, which reflects their generalist foraging behaviors. Specifically, these bees were highly attracted to *S. longispicata* $\times$ *S. farinacea* (big blue salvia) (non-native to FL) (see Fig. S1), which blooms year-round and produces large nectar volumes (Clebsch, 2003). This aligns with the broader concept that generalist pollinators like bumble and carpenter bees adjust foraging preferences and behaviors based on colony needs and environmental nutrient availability (Hendriksma, Toth & Shafir, 2019) and supports the idea that non-native plants can provide benefits to generalist pollinators while more specialized pollinators preferentially forage on plants with which they have evolved (Frankie et al., 2019).

We hypothesized that fully irrigated plants would have increased water uptake and metabolic activity, allowing plants to produce more nectar and pollen. Abiotic factors such as water availability are known to play a direct role in nectar production (Parachnowitsch, Manson & Sletvold, 2019) and previous studies have found that reduced irrigation inputs or drought were associated with decreases in nectar volume per flower or per plant, though there is less known about the effects of drought on pollen production (Descamps et al., 2018; Phillips et al., 2018; Cecala & Wilson-Rankin, 2021; Rering et al., 2020; Kuppler et al., 2021). Moreover, reduced water availability may produce a greater number of nectarless flowers as a way of conserving resources without reducing visitation, as a proportion of nectarless flowers may compel pollinators to visit more flowers per plant (Thakar et al., 2003; Takkis, Tscheulin & Petanidou, 2018). Although irrigation did not affect the amount of nectar and pollen at the flower level in this study, reduced irrigation resulted in lower nectar and pollen quantity at the plot level. One possible explanation for why these effects were detected at the plot but not flower level is that minor reductions in both flower production and resource production per flower under partial irrigation resulted in a significant impact when summed across all flowers in a plot. Thus, while its effects on individual flowers were subtle, irrigation had cumulative effects on both nectar and pollen quantity leading to significant impacts at the plot level.

We also found that irrigation influenced the quality of pollen, specifically total pollen protein content, regardless of plant origin. The differences in pollen quality align with previous studies suggesting that even short-term water limitation can restrict initial storage and accumulation of protein by significantly impacting the transcription of genes involved in protein storage (Begcy et al., 2018). Although we did not quantify consequences at the population level, reductions in nectar and pollen quantity and quality have the potential to affect pollinator fitness and population size especially when relatively minor reductions at a flower or plot level are scaled up to a landscape level and in environments where resources are already scarce.

Contrary to our hypothesis that native plants would respond better to water limitation than non-native plants, we found that both native and non-native plants responded similarly to irrigation treatments. During the experimental period (2021 and 2022), the high distribution of rainfall throughout the year, as reported by the Florida Automated Weather Network (FAWN, 2024) contributed to low water limitation across seasons, and thus likely to reduced effects of the irrigation treatments. Additionally, in this study, the partial irrigation treatment (at 10% volumetric content per hour) was chosen to limit water availability while still sustaining plants and reducing plant mortality. However, this threshold may have been too high for these plants to see an effect of partial irrigation on some of the measured responses. Furthermore, some non-native ornamentals are bred for drought tolerance, especially when cultivated for ornamental use in stressful environments (*e.g*., *G. grandiflora*, *S. longispicata* $\times$ *S. farinacea*). As a result, they may tolerate reduced water inputs as well as native plants. Future studies could control drought conditions more strictly to amplify water limitation effects and assess species-specific responses across non-native plants varying in predicted drought tolerance.

Native and non-native plant species were chosen based on nursery availability, attractiveness to pollinators, and suitability for ornamental landscapes while controlling for floral trait variation between these two groups. However, we could not control for degree of cultivation; non-native plants were more cultivated with a longer history of breeding and were, in a few cases, hybrids. Pollinator responses to these plants may reflect not just region of origin but also degree of cultivation as past studies have found higher pollinator visitation rates to ornamentals more similar to wild-type plants as compared to interspecific hybrids (Hayes et al., 2025). However, our findings are applicable to real-world contexts in which non-native ornamental options are typically highly cultivated. Additionally, non-native plants varied in region of origin with some being from other regions of North America and others being native to other continents, which may have further influenced pollinator responses.

Finally, another limitation of this study is that nectar and pollen sampling was conducted at a single site, which may not fully capture site-specific variations in floral resource production due to microclimatic or soil differences though we expect that relative differences across species would be fairly consistent across sites even if overall resource production differs. Additionally, while we quantified pollen protein content, this alone may not fully explain pollinator preferences, as pollen quality is influenced by a combination of factors and different pollinators have varying nutritional requirements. Nonetheless, our study provides several findings that can influence pollinator garden design and management.

## Conclusions

Our study demonstrates that both plant origin and irrigation shape floral traits and pollinator recruitment, though their effects differ in magnitude and across pollinator guilds. We found strong support for some of our hypotheses: native plants consistently outperformed non-native plants in terms of many floral traits and the recruitment of many pollinator groups. However, both native and non-native species responded similarly to water limitation with resource value of plants (nectar and pollen quantity and pollen protein) being negatively affected by a reduction in irrigation.

To enhance pollinator habitats in domestic gardens, prioritizing native plants is essential, as they generally provide higher floral density, display, and pollen quantity, and attract more native pollinators. However, incorporating non-native plants can still benefit generalist pollinators by supplying additional floral resources and complementing the flowering periods of native species. Limited irrigation (*e.g*., 10% volumetric moisture) may be sufficient to sustain floral resources, including floral display and nectar and pollen per flower, but could still have a subtle yet significant negative effect on the quantity and quality of nectar and pollen at the garden level. Thus, assessing the specific goals of gardening and adjusting management to meet those goals is essential. If the goal is to maximize floral density and display, prioritizing native plants is important, whereas if the focus is to boost nectar and pollen production, irrigation strategies should be carefully considered to create healthy, resource-rich pollinator gardens.

## Supplemental Information

10.7717/peerj.20906/supp-1 Supplemental Information 1 Plant species used in the experiment with their origin, growth form, overall mean plant size as measured in Silva et al. (2024), flower color, and drought tolerance. Detailed botanical and phenological information were already published in Silva et al. (2024).

10.7717/peerj.20906/supp-2 Supplemental Information 2 Summary of generalized linear mixed models (GLMMs) and linear models (LMs) used in the analyses, showing the distributions, fixed effects, and random effects applied to each response variable.

10.7717/peerj.20906/supp-3 Supplemental Information 3 List of insects collected during active sampling on all plots at two sites. Sampling involved a minimum of two people walking down each row and collecting in plots where flowers were present for a period of 5 minutes per plot.

10.7717/peerj.20906/supp-4 Supplemental Information 4 Mean pollinator visitation rates (± SE) across plant species for each pollinator guild.

10.7717/peerj.20906/supp-5 Supplemental Information 5 Raw data of flower abundance and pollinator recruitment across plots, years, and two different sites.

10.7717/peerj.20906/supp-6 Supplemental Information 6 Raw data of insects collected in the field during active sampling across plots, years, and two different sites.

10.7717/peerj.20906/supp-7 Supplemental Information 7 Raw data of nectar volume per flower across plots and years at Plant Science Research and Education Unit (PSREU).

10.7717/peerj.20906/supp-8 Supplemental Information 8 Raw data nectar volume per plot across plots and years at Plant Science Research and Education Unit (PSREU).

10.7717/peerj.20906/supp-9 Supplemental Information 9 Raw data of number of pollen grains per flower across plots at Plant Science Research and Education Unit (PSREU).

10.7717/peerj.20906/supp-10 Supplemental Information 10 Raw data of number of pollen grains per plot across plots at Plant Science Research and Education Unit (PSREU).

10.7717/peerj.20906/supp-11 Supplemental Information 11 Raw data of total pollen protein amount at across plots at Plant Science Research and Education Unit (PSREU).

## Additional Information and Declarations

### Data Availability

The following information was supplied regarding data availability:

The raw data are available in the Supplemental Files.
