# Supplementary material for: Plant origin and irrigation influence floral resource value and pollinator attraction to ornamental plants
Source: PeerJ. 2026 Mar 12;14:e20906. doi: 10.7717/peerj.20906 (PMC12989154; doi:10.7717/peerj.20906)
Supplement: Supplemental Information 1 — Detailed botanical and phenological information were already published in Silva et al. (2024). [file peerj-14-20906-s001.docx]

**SUPPLEMENTAL TABLES**

**Supplemental Table S1.** Plant species used in the experiment with their origin, growth form, overall mean plant size as measured in Silva et al. 2024, flower color, and drought tolerance. Detailed botanical and phenological information were already published in Silva et al. (2024).

| **Species name** | **Category for this study (origin) (see Silva et al., 2024)** | **Mean plant size (cm3) (see Silva et al., 2024)** | **Flower color** | **Drought tolerance** | **References** |
| --- | --- | --- | --- | --- | --- |

| **Herbaceous plants** |
| --- |

| *Bidens alba* | Listed as native to FL. (University of South Florida Plant Atlas, 2025) though possibly only native to S. FL and with considerable debate as to region of origin | 137.5 | White and yellow | High tolerance | (University of South Florida Plant Atlas, 2025) |
| --- | --- | --- | --- | --- | --- |
| *Bidens ferulifolia* ‘BID 16101’ | Cultivar non-native to FL, native to SW U.S. and N. Mexico | 56.0 | Golden yellow | Moderate tolerance | (Proven Winners 2025) |
| *Conradina grandiflora* | Native and endemic to FL | 92.3 | Lavender | High tolerance | (Florida Wildflower Foundation, 2025) |
| *Salvia rosmarinus* ‘Barbeque’1 | Cultivar non-native to FL, native to the Mediterranean. Selected as the non-native pair to *C. grandiflora* as it presents similar morphological traits, is closely related, and there is no non-native *Conradina* *sp.* available in our region | 91.2 | Pale lavender | Moderate tolerance | (Missouri Botanical Garden, 2025) |
| *Coreopsis leavenworthii* | Native to Florida and South Alabama | 103.3 | Yellow and bronze | Moderate tolerance | (Florida Wildflower Foundation, 2025) |
| *Coreopsis* ‘Jethro Tull’ (C. *auriculata ‘Zamfir’ x C. lanceolata ‘Early Sunrise’*) | Native range of *C. auriculata* goes from Virginia to Kentucky and south to Georgia and Louisiana. Native range of *C. lanceolata* includes Florida and most of the U.S. USPP 18,789. | 73.0 | Yellow | Moderate tolerance | (Missouri Botanical Garden, 2025) |
| *Gaillardia pulchella* | Native to the South U.S. Previously considered native to FL | 144.0 | Red /yellow | High tolerance | (Florida Native Plant Society, 2025) |
| *Gaillardia× grandiflora* ‘Arizona Sun’ (*G. aristata* x *G. pulchella*) | Hybrid cultivar with parents’ native to various parts of the U.S. Parent species of this hybrid are *G. aristata* (native range from North Dakota to Colorado and west to California and British Columbia) and *G. pulchella.* | 76.6 | Red, orange, and yellow | Moderate tolerance | (Missouri Botanical Garden, 2025) |
| *Monarda punctata* | Native to Florida and the Eastern U.S. | 155.4 | Purple-spotted | Moderate tolerance | (Florida Native Plant Society, 2025) |
| *Monarda didyma* ‘Pardon My Pink’ | Cultivar non-native to FL. Hybridized using *M. didyma* ‘Acrade’ and *M. didyma* ‘Achall’. *M. didyma* is native to much of the eastern U.S. south to Georgia. USPP 24,244. | 52.4 | Fuchsia-pink | Low tolerance | (Missouri Botanical Garden 2025) |
| *Salvia azurea* | Native to FL and Central/Eastern U.S. | 130.0 | Blue | High tolerance | (Florida Native Plant Society, 2025) |
| *Salvia longispicata × S. farinacea* ‘PAS1246577’ | Hybrid cultivar non-native to FL. *S. longispicata* is native to southwestern Mexico while *S. farinacea* is native to TX, NM, and OK. | 145.0 | Purple-blue | High tolerance | (Chicago Botanic Garden, 2025) |
| *Scutellaria arenicola* | Native to FL | 83.0 | Lavender white | Moderate tolerance | (Florida Wildflower Foundation, 2025) |
| *Scutellaria javanica* | Non-native to FL, native to SE Asia | 73.8 | Dark purple | Low to moderate tolerance | (North Carolina State University Plant Toolbox, 2025) |

| **Woody plants** |
| --- |

| *Hibiscus grandiflorus* | Native to FL and SE U.S. wetlands | 166.1 | Light pink | Low tolerance | (Florida Native Plant Society, 2025) | |
| --- | --- | --- | --- | --- | --- | --- |
| *Hibiscus syriacus* ‘SHIMCR1’ | Cultivar non-native to FL, native to Asia. A cross between *H. syriacus* ‘Kwangmyung’ and *H. syriacus* ‘Samchulli’. USPP 26,222 | 67.7 | Pink withred eye | Moderate tolerance | (Missouri Botanical Garden, 2025) | |
| *Ilex glabra* | Native to FL and E. coastal plain U.S. | 121.9 | White | Moderate to high tolerance | (Florida Native Plant, Society 2025) |  |
| *Ilex cornuta* ‘Dwarf Burford’ | Cultivar non-native to FL, native to China and Korea. | 165.0 | White | High tolerance | (Missouri Botanical Garden, 2025) | |
| *Viburnum obovatum* | Native to FL and SE U.S. floodplains | 190.0 | White | Moderate to high tolerance | (Florida Native Plant Society, 2025) | |
| *Viburnum suspensum* | Non-native to FL, native to Japan | 114.0 | White | Moderate tolerance | (Missouri Botanical Garden, 2025g, Everde Growers, 2025) | |
