# Supplementary material for: Plant origin and irrigation influence floral resource value and pollinator attraction to ornamental plants
Source: PeerJ. 2026 Mar 12;14:e20906. doi: 10.7717/peerj.20906 (PMC12989154; doi:10.7717/peerj.20906)
Supplement: Supplemental Information 2 [file peerj-14-20906-s002.docx]

**SUPPLEMENTAL TABLES**

**Supplemental Table 2.** Summary of generalized linear mixed models (GLMMs) and linear models (LMs) used in the analyses, showing the distributions, fixed effects, and random effects applied to each response variable.

| **Response variable** | **Distribution / model type** | **Fixed effects** | **Random effects** |
| --- | --- | --- | --- |
| Flower density per plot per sampling day | GLMM: Negative binomial | Origin, Irrigation, Origin × Irrigation | (1 \| Site/Plot) + (1 \| Year) |
| Floral display per plot per sampling day | GLMM: Tweedie | Origin, Irrigation, Origin × Irrigation | (1 \| Site/Plot) + (1 \| Year) |
| Nectar volume per flower per plot per sampling day | GLMM: Gamma | Origin, Irrigation, Origin × Irrigation | (1 \| Plot) + (1 \| sampler) |
| Pollen quantity per flower per plot | GLMM: Negative binomial | Origin, Irrigation, Origin × Irrigation | (1 \| Plot) |
| Standardized pollen protein per flower | LM: Normal | Origin, Irrigation, Origin × Irrigation | — |
| Total nectar volume per plot per sampling day | GLMM: Tweedie | Origin, Irrigation, Origin × Irrigation | (1 \| Site/Plot) + (1 \| Year) |
| Total pollen quantity per plot per sampling day | GLMM: Negative binomial | Origin, Irrigation, Origin × Irrigation | (1 \| Site/Plot) + (1 \| Year) |
| Total pollinator visits per plot per sampling period | GLMM: Negative binomial | Origin, Irrigation, Origin × Irrigation | (1 \| Site/Plot) + (1 \| Year) |
| Pollinator visits by group (bumble bees, carpenter bees, other native bees, wasps, butterflies/moths, other insects) | GLMM: Negative binomial | Origin, Irrigation, Origin × Irrigation | (1 \| Site/Plot) + (1 \| Year) |
| Pollinator visits by group (honey bees, flies) | Zero-inflated GLMM: Negative binomial | Origin, Irrigation, Origin × Irrigation | (1 \| Site/Plot) + (1 \| Year) |
| Shannon diversity index of pollinator community | GLMM: Normal (log-transformed) | Origin, Irrigation, Origin × Irrigation | (1 \| Site/Plot) + (1 \| Year) |
| Pollinator richness per plot per sampling day | GLMM: Negative binomial | Origin, Irrigation, Origin × Irrigation | (1 \| Site/Plot) + (1 \| Year) |
