# Supplementary material for: Plant origin and irrigation influence floral resource value and pollinator attraction to ornamental plants
Source: PeerJ. 2026 Mar 12;14:e20906. doi: 10.7717/peerj.20906 (PMC12989154; doi:10.7717/peerj.20906)
Supplement: Supplemental Information 3 — Sampling involved a minimum of two people walking down each row and collecting in plots where flowers were present for a period of 5 minutes per plot. [file peerj-14-20906-s003.docx]

**SUPPLEMENTAL TABLES**

**Supplemental Table 3.** List of insects collected during active sampling on all plots at two sites. Sampling involved a minimum of two people walking down each row and collecting in plots where flowers were present for a period of 5 minutes per plot.

|  | | | |
| --- | --- | --- | --- |
| **Group and Order** | **Family** | **Species name** | **Author** |
| **Honey Bees** |  |  |  |
| Hymenoptera | Apidae | *Apis mellifera* | Linnaeus |
| **Bumble bees** | | | |
| Hymenoptera | Apidae | *Bombus impatiens* | (Cresson) |
| Hymenoptera | Apidae | *Bombus pensilvanicus* | (De Geer, 1773) |
| **Carpenter bees** | | | |
| Hymenoptera | Apidae | *Xylocopa micans* | Lepeletier, 1841 |
| Hymenoptera | Apidae | *Xylocopa virginica* | (Linnaeus) |
| **Other bees** | | | |
| Hymenoptera | Apidae | *Habrapoda laboriosa* | (Fabricius, 1804) |
| Hymenoptera | Apidae | *Melissodes communis* | [Cresson, 1878](https://en.wikipedia.org/wiki/Ezra_Townsend_Cresson) |
| Hymenoptera | Apidae | *Melissodes comptoides* | Robertson, 1898 |
| Hymenoptera | Apidae | *Melissodes manipularis* | Smith, 1854 |
| Hymenoptera | Apidae | *Melissodes obliqua* | (Say, 1837) |
| Hymenoptera | Apidae | *Nomada fervida* | [Smith, 1854](https://en.wikipedia.org/wiki/Frederick_Smith_(entomologist)) |
| Hymenoptera | Apidae | *Nomada vegana* | [Cockerell, 1903](https://en.wikipedia.org/wiki/Theodore_Dru_Alison_Cockerell) |
| Hymenoptera | Apidae | *Svastra atripes* | [(Cresson, 1872)](https://en.wikipedia.org/wiki/Ezra_Townsend_Cresson) |
| Hymenoptera | Apidae | *Svastra obliqua* | [(Say, 1837)](https://en.wikipedia.org/wiki/Thomas_Say) |
| Hymenoptera | Apidae | *Triepeolus concavus* | [(Cresson, 1878)](https://en.wikipedia.org/wiki/Ezra_Townsend_Cresson) |
| Hymenoptera | Apidae | *Triepeolus lunatus lunatus* | [(Say, 1824)](https://en.wikipedia.org/wiki/Thomas_Say) |
| Hymenoptera | Apidae | *Triepeolus remigatus* | [(Fabricius, 1804)](https://en.wikipedia.org/wiki/Johan_Christian_Fabricius) |
| Hymenoptera | Halictidae | *Agapostemon splendens* | (Lepeletier, 1841) |
| Hymenoptera | Halictidae | *Halictus confusus* | [Smith, 1853](https://en.wikipedia.org/wiki/Frederick_Smith_(entomologist)) |
| Hymenoptera | Halictidae | *Halictus poeyi* | Lepeletier, 1841 |
| Hymenoptera | Halictidae | *Halictus rubicundus* | [(Christ, 1791)](https://en.wikipedia.org/wiki/Johann_Ludwig_Christ) |
| Hymenoptera | Halictidae | *Lasioglossum alachuense* | Mitchell, 1960 |
| Hymenoptera | Halictidae | *Lasioglossum apopkense* | (Robertson, 1892) |
| Hymenoptera | Halictidae | *Lasioglossum creberrimun* | (Smith, 1853) |
| Hymenoptera | Halictidae | *Lasioglossum floridanum* | Robertson, 1892 |
| Hymenoptera | Halictidae | *Lasioglossum longifrons* | (Baker, 1906) |
| Hymenoptera | Halictidae | *Lasioglossum pectorale* | (Smith, 1853) |
| Hymenoptera | Halictidae | *Lasioglossum puteulanum* | Gibbs, 2009 |
| Hymenoptera | Halictidae | *Sphecodes mandibularis* | Cresson, 1872 |
| Hymenoptera | Megachilidae | *Coelioxys modestus* | Smith, 1854 |
| Hymenoptera | Megachilidae | *Megachile albitarsis* | Cresson, 1872 |
| Hymenoptera | Megachilidae | *Megachile integra* | Cresson, 1878 |
| Hymenoptera | Megachilidae | *Megachile mendica* | Cresson, 1878 |
| Hymenoptera | Megachilidae | *Megachile policaris* | Say, 1831 |
| Hymenoptera | Megachilidae | *Megachile pruina pruina* | Smith, 1853 |
| Hymenoptera | Megachilidae | *Megachile pseudobrevis* | Mitchell, 1934 |
| **Wasps** | | | |
| Hymenoptera | Ichneumonidae | Ichneumonidae 1 | Latreille, 1802 |
| Hymenoptera | Pteromalidae | Pteromalidae 1 | [Dalman, 1820](https://en.wikipedia.org/wiki/Johan_Wilhelm_Dalman) |
| Hymenoptera | Vespidae | *Euodynerus castigatus* | (de Saussure, 1853) |
| Hymenoptera | Vespidae | *Euodynerus hidalgo* | (de Saussure, 1857) |
| Hymenoptera | Vespidae | *Pachodynerus erynnis* | (Lepeletier, 1841) |
| Hymenoptera | Vespidae | *Polistes exclamans* | [Viereck, 1906](https://en.wikipedia.org/wiki/Henry_Lorenz_Viereck) |
| Hymenoptera | Vespidae | *Polistes fuscatus* | (Fabrizio , 1793) |
| Hymenoptera | Vespidae | *Polistes major* | Palisot de Beauvois, 1818 |
| Hymenoptera | Vespidae | *Polystes sp.* | Linnaeus, 1767 |
| Hymenoptera | Vespidae | *Pseudodynerus quadrisectus* | Say, 1837 |
| Hymenoptera | Vespidae | *Stenodynerus fundatiformis* | (Saussure, 1856) |
| Hymenoptera | Vespidae | *Zethus spinipes* | Say, 1837 |
| Hymenoptera | Thynnidae | *Myzinum carolinianum* | (Fabricius, 1805) |
| Hymenoptera | Thynnidae | *Myzinum maculatum* | (Fabricius, 1793) |
| Hymenoptera | Thynnidae | *Myzinum obscurum* | (Fabricius, 1793) |
| Hymenoptera | Thynnidae | *Myzinum quinquecinctum* | (Fabricius, 1775) |
| Hymenoptera | Pompilidae | *Paracyphononyx funereus* | (Lepeletier, 1845) |
| Hymenoptera | Pompilidae | *Pepsis sp.* |  |
| Hymenoptera | Pompilidae | *Poecilopompilus algidus* | (Smith, 1855) |
| Hymenoptera | Scoliidae | *Colpa octomaculata hermione* | (Say, 1823) |
| Hymenoptera | Scoliidae | *Dielis plumipes fossulana* | (Drury, 1770) |
| Hymenoptera | Scoliidae | *Pygodasis quadrimaculata* | (Fabricius, 1793) |
| Hymenoptera | Scoliidae | *Scolia dubia* | (Say, 1837) |
| Hymenoptera | Scoliidae | *Scolia nobilitata nobilitata* | (Fabricius, 1805) |
| Hymenoptera | Formicidae | Formicidae 1 |  |
| Hymenoptera | Sphecidae | *Chalybion californicum* | (de Saussure, 1867) |
| Hymenoptera | Sphecidae | *Prionyx parkeri* | Bohart & Menke, 1963 |
| Hymenoptera | Sphecidae | *Sphex dorsalis* | Fargeau, 1845 |
| Hymenoptera | Sphecidae | *Sphex ichneumoneus* | (Linnaeus, 1758) |
| Hymenoptera | Crabronidae | *Bembix americana* | Fabricius, 1793 |
| Hymenoptera | Crabronidae | *Cerceris rufopicta* | Smith, 1856 |
| Hymenoptera | Crabronidae | *Cerceris bicornuta* | Guérin-Méneville, 1844 |
| Hymenoptera | Crabronidae | *Cerceris fumipennis* | Say, 1837 |
| Hymenoptera | Crabronidae | *Philanthus ventilabris* | Fabricius, 1798 |
| Hymenoptera | Crabronidae | *Tachytes guatemalensis* | Cameron, 1889 |
| **Flies** | | | |
| Diptera | Tipulidae | Tipulidae 1 |  |
| Diptera | Bibionidae | Bibionidae 1 |  |
| Diptera | Asiilidae | *Diomites misellus* | Loew, 1866 |
| Diptera | Bombyliidae | *Poecilanthrax lucifer* | (Fabricius, 1775) |
| Diptera | Mydidae | *Phyllomydas parvulus* | (Westwood, 1841) |
| Diptera | Conopidae | *Physocephala marginata* | (Say, 1823) |
| Diptera | Conopidae | *Physocephala sagittaria* | (Say, 1823) |
| Diptera | Tephritidae | Tephritidae 1 |  |
| Diptera | Ulidiidae | Ulidiidae 1 |  |
| Diptera | Lauxaniidae | Lauxaniidae 1 |  |
| Diptera | Muscidae | Muscidae 1 |  |
| Diptera | Sarcophagidae | *Sarcophagidae* |  |
| Diptera | Syrphidae | *Allograpata exotica* | (Wiedemann , 1830) |
| Diptera | Syrphidae | *Palpada sp.* | Macquart, 1854 |
| Diptera | Syrphidae | *Syritta flaviventris* | Macquart, 1842 |
| Diptera | Syrphidae | *Toxomerus marginatus* | (Say, 1823) |
| Diptera | Syrphidae | *Tropidia sp.* | Meigen, 1822 |
| **Butterflies and moths** | | | |
| Lepidoptera | Crambidae | *Pyrausta tyralis* | (Guenée, 1854) |
| Lepidoptera | Erebidae | *Syntomeida ipomoeae* | (Harris, 1839) |
| Lepidoptera | Noctuidae | *Marimatha nigrofimbria* | (Guenée, 1852) |
| Lepidoptera | Noctuidae | *Spodoptera sp.* | Guenée, 1852 |
| Lepidoptera | Noctuidae | *Spragueia onagrus* | (Guenée, 1852) |
| Lepidoptera | Papilionidae | *Papilo polixenes* | (Fabricius, 1775) |
| Lepidoptera | Hesperidae | *Anatrytone logan* | (W.H. Edwards, 1863) |
| Lepidoptera | Hesperidae | *Atalopedes campestris* | Boisduval, 1852 |
| Lepidoptera | Hesperidae | *Burnsius albescens* | Grishin, 2022 |
| Lepidoptera | Nymphalidae | *Agraulis vanillae* | Linnaeus |
| **Other insects** | | | |
| Hemiptera | Alydidae | Alydidae 1 |  |
| Hemiptera | Alydidae | Alydidae 2 |  |
| Hemiptera | Cercopidae | Cercopidae 1 |  |
| Hemiptera | Coreidae | Coreidae 1 |  |
| Hemiptera | Lygaeidae | Lygaeidae 1 |  |
| Hemiptera | Miridae | Miridae 1 |  |
| Hemiptera | Pentatomidae | Pentatomidae 1 |  |
| Hemiptera | Pentatomidae | Pentatomidae 2 |  |
| Hemiptera | Pyrrhocoridae | Pyrrhocoridae 1 |  |
| Hemiptera |  | Hemiptera 1 |  |
| Coleoptera | Cantharidae | *Chauliognathus marginatus* | (Fabricius, 1775) |
| Coleoptera | Scarabeidae | *Conitis nitida* | (Linnaeus, 1758) |
| Coleoptera | Scarabeidae | *Euphoria sepulcralis* | (Fabricius, 1801) |
| Coleoptera | Mordellidae | *Mordella atrata* | Melsheimer, 1845 |
| Coleoptera | Tenebrionidae | Tenebrionidae 1 |  |
| Coleoptera | Coccinelidae | *Coccinella sp.* | Linnaeus, 1758 |
| Coleoptera | Coccinellidae | *Cycloneda sp.* |  |
| Coleoptera | Curculionidae | *Chalcodermus aeneus* | Boheman, 1837 |
| Coleoptera | Curculionidae | Curculionidae 1 |  |
| Coleoptera | Chrysomelidae | *Altica chalybea* | Illiger, 1807 |
