# Supplementary figures and images for: Plant origin and irrigation influence floral resource value and pollinator attraction to ornamental plants
Source: PeerJ. 2026 Mar 12;14:e20906. doi: 10.7717/peerj.20906 (PMC12989154; doi:10.7717/peerj.20906)

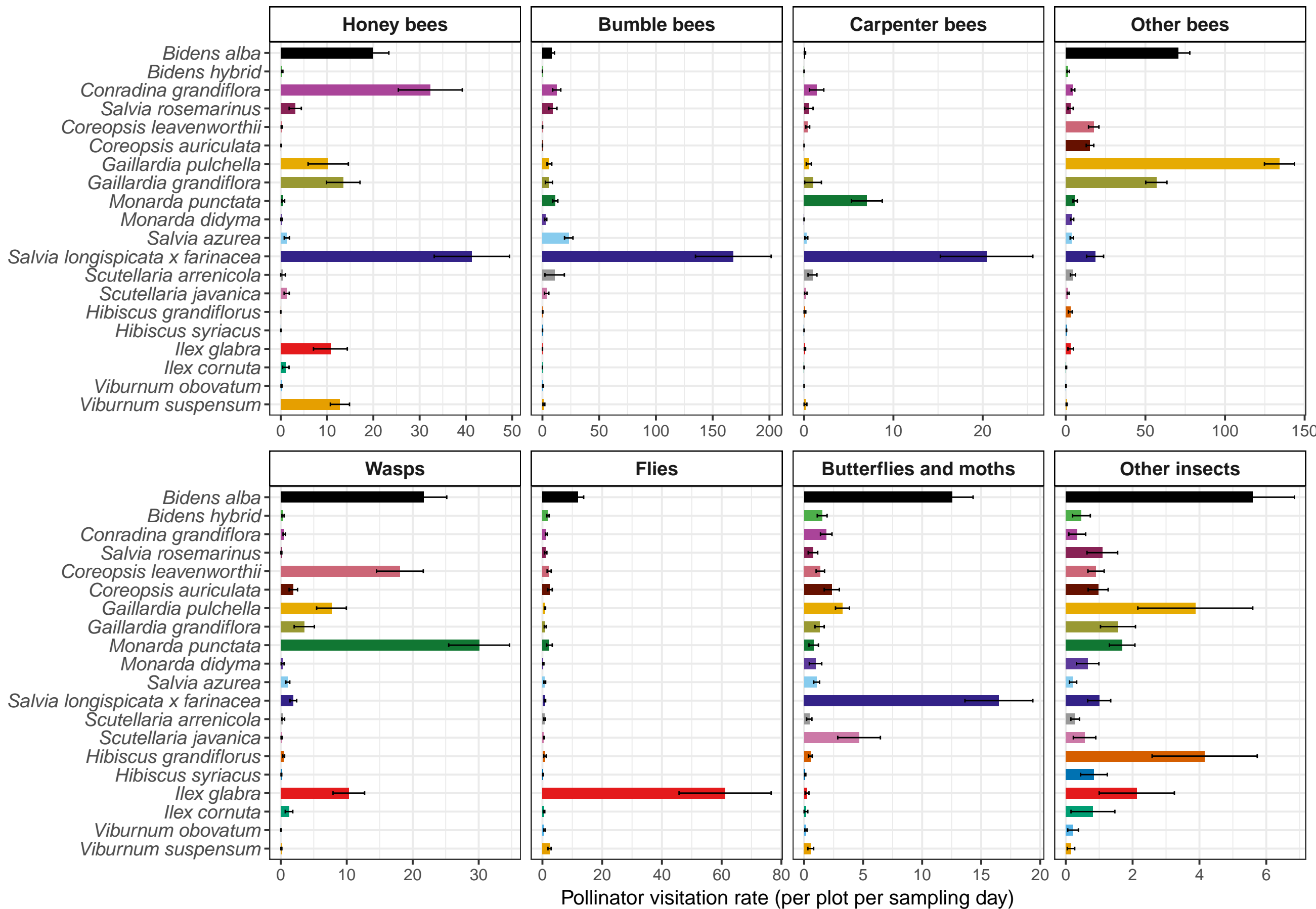

Supplement: Supplemental Information 4 [file peerj-14-20906-s004.pdf]
